# Supplementary material for: Mechanisms governing the pioneering and redistribution capabilities of the non-classical pioneer PU.1
Source: Nat Commun. 2020 Jan 21;11:402. doi: 10.1038/s41467-019-13960-2 (PMC6972792; doi:10.1038/s41467-019-13960-2)
Supplement: Supplementary file 7 — Source data [file 41467_2019_13960_MOESM7_ESM.zip › Source_Data/Figure6/Figure6B_MotifScanOutput/ETS-specific/homerResults/motif15.similar.html]

motif15

## Information for motif15

A
T
G
C
A
G
C
T
C
G
T
A
C
G
T
A
A
C
G
T
A
T
G
C
C
T
G
A
A
T
C
G
G
C
A
T
  
Reverse Opposite:  

C
G
T
A
A
T
G
C
G
A
C
T
A
T
C
G
C
G
T
A
A
C
G
T
C
G
A
T
C
T
G
A
T
A
C
G
  

|  |  |
| --- | --- |
| p-value: | 1e-74 |
| log p-value: | -1.711e+02 |
| Information Content per bp: | 1.670 |
| Number of Target Sequences with motif | 1043.0 |
| Percentage of Target Sequences with motif | 13.63% |
| Number of Background Sequences with motif | 2819.3 |
| Percentage of Background Sequences with motif | 6.99% |
| Average Position of motif in Targets | 100.1 +/- 56.2bp |
| Average Position of motif in Background | 98.2 +/- 60.3bp |
| Strand Bias (log2 ratio + to - strand density) | 0.0 |
| Multiplicity (# of sites on avg that occur together) | 1.08 |
| Motif File: | file (matrix) reverse opposite |

### Similar de novo motifs found

|  |  |  |  |  |  |  |  |
| --- | --- | --- | --- | --- | --- | --- | --- |
| Rank | Match Score | Redundant Motif | P-value | log P-value | % of Targets | % of Background | Motif file |
| 1 | 0.828 | A C G T C G T A C G T A A G C T A G T C C G T A A C T G | 1e-45 | -105.114835 | 18.05% | 11.86% | motif file (matrix) |
| 2 | 0.838 | A G T C G T A C C G T A C G T A A C G T T A G C T C A G A T C G | 1e-22 | -52.051533 | 5.90% | 3.40% | motif file (matrix) |
| 3 | 0.817 | C G A T A T G C G A T C T A C G C G T A A G C T C G A T A C T G C A T G G T A C | 1e-18 | -42.009849 | 4.30% | 2.39% | motif file (matrix) |
